# Is It Speech or Song? Effect of Melody Priming on Pitch Perception of Modified Mandarin Speech

**DOI:** 10.3390/brainsci9100286

**Published:** 2019-10-22

**Authors:** Chen-Gia Tsai, Chia-Wei Li

**Affiliations:** 1 Graduate Institute of Musicology, National Taiwan University, Taipei 106, Taiwan; tsaichengia@ntu.edu.tw; 2 Neurobiology and Cognitive Science Center, National Taiwan University, Taipei 106, Taiwan; 3 Department of Radiology, Wan Fang Hospital, Taipei Medical University, Taipei 116, Taiwan

**Keywords:** melody perception, tonal language, inferior frontal gyrus, priming effect

## Abstract

Tonal languages make use of pitch variation for distinguishing lexical semantics, and their melodic richness seems comparable to that of music. The present study investigated a novel priming effect of melody on the pitch processing of Mandarin speech. When a spoken Mandarin utterance is preceded by a musical melody, which mimics the melody of the utterance, the listener is likely to perceive this utterance as song. We used functional magnetic resonance imaging to examine the neural substrates of this speech-to-song transformation. Pitch contours of spoken utterances were modified so that these utterances can be perceived as either speech or song. When modified speech (target) was preceded by a musical melody (prime) that mimics the speech melody, a task of judging the melodic similarity between the target and prime was associated with increased activity in the inferior frontal gyrus (IFG) and superior/middle temporal gyrus (STG/MTG) during target perception. We suggest that the pars triangularis of the right IFG may allocate attentional resources to the multi-modal processing of speech melody, and the STG/MTG may integrate the phonological and musical (melodic) information of this stimulus. These results are discussed in relation to subvocal rehearsal, a speech-to-song illusion, and song perception.

## 1. Introduction

Tonal languages are characterized by the use of lexical tones for distinguishing lexical semantics. Lexical tones include distinct level pitches and pitch-glide patterns. Owing to pitch variation, spoken utterances in tonal languages are rich in melody and sometimes comparable to music. Related to this, it has been acknowledged that tonal languages and music share similarity in the perceptual-cognitive processing of pitch. Recognition of lexical tones relies on relations between successive pitches [1,2,3], and thus the underlying neural substrates partially overlapped with those underlying recognition of musical pitch intervals [4]. Evidence of similarity between spoken utterances of tonal languages and music also comes from traditional music. The distinction between speech and song is blurred in many genres of Chinese musical theater. For example, it has been suggested that eight oral delivery types in Cantonese opera occupy different positions on a speech-music spectrum according to their tonal features, rhythmic features, and instrumental accompaniment [5]. In Chinese opera, a sung utterance may be perceived as somewhat speech-like because of high congruency between its musical melody and lexical tones. On the other hand, the pitches of a spoken utterance could be embedded into the musical scale provided by the accompaniment music. Using this musical scale as a tonal schema, listeners may perceive this utterance as song.

The fact that the tonal context provided by an instrumental accompaniment could perceptually transform speech of a tonal language to song raises a possibility that the listener could perceive the melody of a spoken utterance as a musical melody when he/she is primed by appropriate musical cues. In the present study, we reported a novel priming effect of musical melody on pitch perception of spoken Mandarin utterances. The target was a speech-like stimulus. The melody of this target was mimicked by a musical melody, which served as the prime. When the listener was primed by this musical melody, he/she tended to perceive the target as song.

Previous studies have reported that acoustically identical English utterances can be perceived as either speech or song. Deutsch and colleagues found that when a spoken English phrase was repeated several times, listeners were likely to perceive this phrase as song [6]. The authors hypothesized that exposure to repetition of a speech fragment may be associated with greater activity in the neural substrates of pitch processing, relative to the condition in which a spoken phrase was presented once. Moreover, this repetition effect may result in a re-evaluation of prosodic features of this spoken phrase [7]. These two hypotheses for the speech-to-song illusion were supported by previous neuroimaging studies demonstrating that (1) the effect of perceiving a spoken phrase as song via repetition localized to the right mid-posterior superior temporal sulcus (STS) and middle temporal gyrus (MTG) implicated in pitch processing [8,9], and (2) the subjective vividness of the speech-to-song illusion was positively correlated with activity in a left frontotemporal loop implicated in evaluation of linguistic prosody. This left frontotemporal loop comprises the inferior frontal gyrus (IFG), frontal pole, and temporal pole [9]. 

Using functional magnetic resonance imaging (fMRI), the present study aimed at specifying the neural underpinnings of the perceptual transformation from Mandarin speech-like utterances to song across a musical prime that mimics the melody of speech. In light of the aforementioned studies of the speech-to-song illusion in English, we hypothesized that the effect of melody priming on the pitch processing of Mandarin speech-like utterances would be associated with increased activity in the IFG, which may contribute to the cognitive processes for attending to the melodic features of speech and for comparing speech with the musical prime. Specifically, the pars triangularis of the right IFG (IFGtri) seems to play a prominent role in evaluation of prosodic information of speech [10,11,12]. We expected to observe greater activity within the right IFGtri during listening to Mandarin speech-like utterances preceded by a melody prime, compared to listening to the same stimulus without melody priming. In addition, we hypothesized that the anterior insula and supplementary motor area (SMA) implicated in subvocal rehearsal [13] may co-activate with the right IFGtri because participants may engage subvocal rehearsal strategies for encoding the melodic features of Mandarin speech-like utterances.

In addition to attention control and sensorimotor mechanisms, the present study was also expected to shed new light on the perceptual processing of song. Sammler and colleagues employed a functional magnetic resonance adaptation paradigm to identify the neural correlates of binding lyrics and tunes in unfamiliar song [14]. Results revealed that the left mid-posterior STS showed an interaction of the adaptation effects for lyrics and tunes. The authors suggested that this region may contribute to an integrative processing of lyrics and tunes. Alonso and colleagues reported that binding lyrics and tunes for the encoding of new songs was associated with the involvement of the bilateral mid-posterior MTG [15]. In the present study, a Mandarin speech-like utterance could be perceived as song when it was preceded by a musical melody mimicking the melody of the utterance. We hypothesized that melody priming would lead to increased activity in the STS/MTG implicated in binding lyrics and tunes during song perception.

## 2. Materials and Methods

### 2.1. Participants

Twenty native Mandarin speakers (age range 20–43 years; six males) participated in the fMRI experiment, in which three fMRI scanning runs for the present linguistic study (focusing on a tonal language) alternated with two fMRI scanning runs for a musical study (focusing on symphonies and concertos). This design was used to minimize affective habituation that could occur with repeated exposure to the same emotional music. The selection and recruitment of participants are mentioned in the next paragraph. Other methods and results of the musical study are not mentioned further in this paper.

Participants were recruited via a public announcement on the internet, which stated the requirement of a high familiarity with Western classical music. In a pre-scan test, volunteers were asked to write down their feelings in response to the musical stimuli in the musical study. Eight musical stimuli with duration of 30 s were presented in a fixed order. After listening to each 30-s stimulus, the volunteers were asked to write down their feelings in response to the passage just before the theme recurrence and their feelings in response to the theme recurrence. They were also asked to explain their feelings in terms of musical features. The first author of this article (a musicologist) selected participants for the fMRI experiment according to the following inclusion criteria: (1) more than five passages just prior to the theme recurrence evoked his/her anticipation; (2) the recurrence of more than five themes evoked a feeling of resolution; and (3) their feelings were appropriately explained in terms of musical features for more than five excerpts. Thirty-one adult volunteers completed this questionnaire. Twenty-seven volunteers met our screening criteria. Twenty of them completed the fMRI experiment. They were free from neurological, psychiatric, or auditory problems. Fifteen participants studied musical instruments for six years or more. The participants were compensated with approximately 16 USD after the completion of fMRI scan.

In the present linguistic study, participants were excluded from analyses of fMRI data if they were unable to discriminate between matched and mismatched trials at better than chance levels (see 2.5. Data Analysis). A female participant was excluded in this way. The data of another female participant were discarded because of incomplete behavioral data acquisition during the fMRI session. Thus, the final sample included in fMRI analyses consisted of 18 adults (mean age = 27.1 years; SD = 6.5 years; mean experience in most experienced instrument = 8.9 years; SD = 4.2 years; six males). Written informed consent was obtained from each participant prior to participation in the study. All research procedures were performed in accordance with a protocol approved by the Institutional Review Board of National Taiwan University (201611HM008). This study was conducted in accordance with the Declaration of Helsinki.

### 2.2. Stimuli

Auditory stimuli were noise, linguistic stimuli, and musical stimuli. The noise stimulus was white noise with a duration of 1.6 s. The duration of each linguistic and musical stimulus was 1.9–2.4 s. The linguistic stimuli were spoken sentences in Mandarin. Each sentence contained six characters. A female broadcaster was invited to recite 65 sentences with flat affect and natural prosody. These materials were recorded and saved as digital sound files.

**Figure 1:**
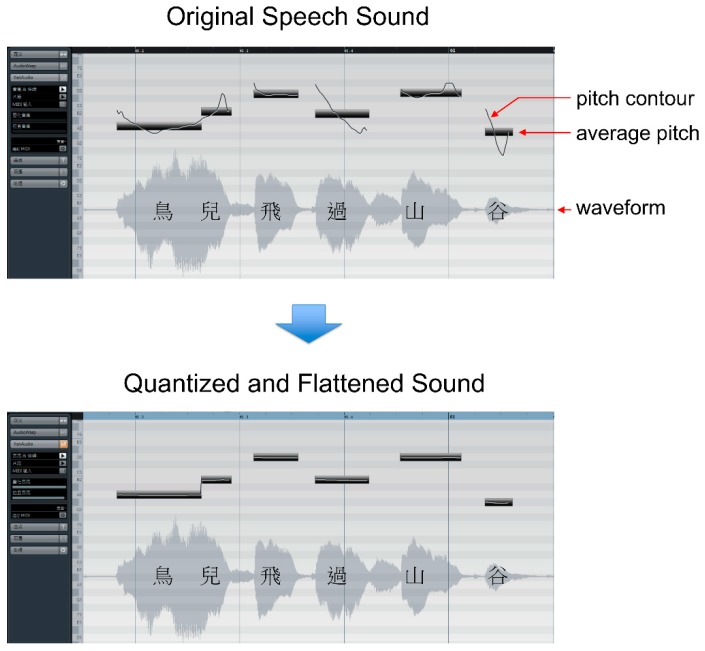
The first and second steps (quantizing and flattening) of pitch adjustment of spoken utterances using Cubase (Steinberg Media Technologies GmbH by VeriSign, Inc.) The pitches were adjusted to match the nearest note in the chromatic musical scale (quantizing). All pitch glides of the spoken utterances were flattened by approximately 95% (flattening).

To generate stimuli that can be perceived as either speech or song, three steps of pitch adjustment were applied on the spoken utterances. These steps are similar to the auto-tune processes used to “songify” news reports or any normal speech. The first step was “quantizing”; the pitches were adjusted to match the nearest note in the chromatic musical scale. The second step was “flattening”; pitch glides of these spoken utterances were flattened by approximately 95%. Figure 1 illustrates the effects of quantizing and flattening on a spoken Mandarin sentence. Third, the first author adjusted the melody of each utterance to match the C major or B-flat major scales by transposing some pitches by a semitone. These three steps were carried out using Cubase (Steinberg Media Technologies GmbH by VeriSign, Inc., HH, Germany) The modified spoken utterances can be perceived as speech because of high congruency between their pitch contours and lexical tones. They can also be perceived as song because their pitches can be embedded into the musical scale. Among the 65 utterances, 50 utterances for the scanning session and 6 utterances for the training session were selected by the first author.

All musical stimuli were melodies containing 6–10 notes, with the pitch ranging from E3 to F4 (fundamental frequency 164.8–349.2 Hz). There were three types of musical stimuli: Match, Mismatch, and Melody-Variation types. The musical stimuli of the Match type were melodies extracted from the linguistic stimuli using “MIDI extraction” in Cubase. As a result, each melody of the Match type closely resembles the melody of a linguistic stimulus. Each melody of the Mismatch type was generated from each melody of the Match type by elevating or lowering the pitches of 4–7 notes by 2–9 semitones while keeping the rhythm and tonality unchanged. The musical stimuli of the Melody-Variation type were paired melodies; the first melody (prime) was composed by the first author of this article, and the second melody (target) was generated from the first melody by elevating or lowering the pitches of 4–7 notes by 2–9 semitones while keeping the rhythm and tonality unchanged. All musical stimuli were in the C major or B-flat major tonalities and generated with a virtual musical instrument named “oboe” using Reason 7.0 (Propellerhead Inc., STH, Sweden).

**Figure 2:**
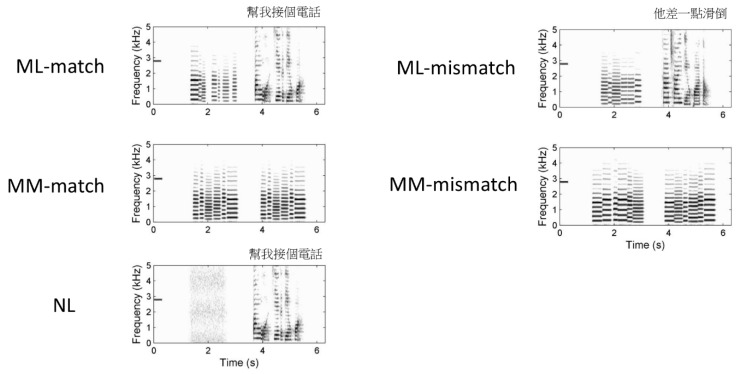
Examples of spectrograms of the stimuli in the five conditions. (ML-match: melody-language-match; ML-mismatch: melody-language-mismatch; MM-match: melody-melody-match; MM-mismatch: melody-melody-mismatch; NL: noise-language).

There were five conditions in this study, as depicted in Figure 2. The experimental condition was melody-language-match (ML-match) condition, in which the prime was a musical stimulus mimicking the melody of the target linguistic stimulus. In the noise-language (NL) condition, the prime was noise, and the target was a linguistic stimulus. In the melody-melody-match (MM-match) condition, the prime and the target were the same musical melody. The NL and MM-match conditions were two control conditions of this study. Compared to NL, ML-match additionally demanded attention to the melodic features of speech, pitch processing, and tonal working memory. Both ML-match and MM-match demanded pitch processing and tonal working memory, as participants were asked to judge the melodic similarity between the prime and target. Compared to MM-match, ML-match additionally demanded selective allocation of attention to the melodic features of speech. For audio examples of the stimuli for NL and ML-match, see Supplementary Materials.

The stimuli of the Mismatch and Melody-Variation types were used in the melody-language-mismatch (ML-mismatch) and melody-melody-mismatch (MM-mismatch) conditions, respectively. In the ML-mismatch condition, the prime was a musical melody that mismatched the melody of the target linguistic stimulus. In the MM-mismatch condition, the prime was a musical melody that mismatched the target musical melody. There were 20 trials in each of the experimental and control conditions, whereas there were 10 trials in each of the two mismatch conditions. The fMRI data for the two mismatch conditions were not analyzed. The target stimuli for ML-match and NL were counter-balanced across participants; the spoken utterances in ML-match were used as the target stimuli in NL for the other 10 participants, and vice versa. 

### 2.3. Procedure

The current study included a training session and a scanning session, which were separated by 5–10 min. In the training session, the participants were trained outside the MRI scanner room to familiarize themselves with the tasks. The experimenter explained to each participant with a PowerPoint presentation and sound examples that the melody of a Mandarin utterance can be compared to a musical melody. For demonstration, the experimenter rated the melodic similarity between a target and a prime on a 4-point Likert scale for five trials (one trial for each condition) by pressing a button (a right-most button for “very similar”, a right button for “slightly similar”, a left button for “slightly dissimilar”, and a left-most button for “very dissimilar”). In a similar manner, the participant practiced six trials, including two trials for ML-match and one trial for each of the other four conditions. This training session lasted approximately 15 min. None of the stimuli used in the training session were presented in the scanning session.

**Figure 3:**
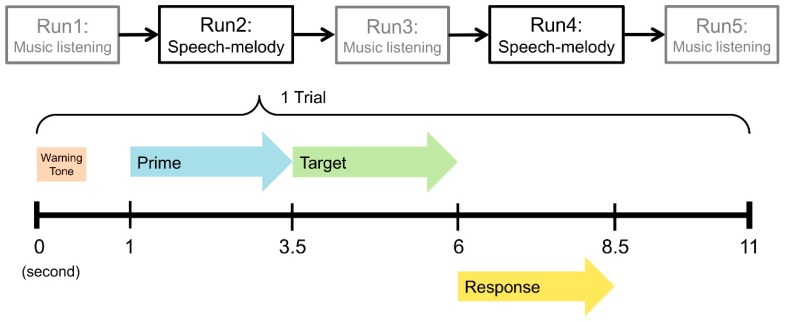
Schematic description of the procedure of the functional magnetic resonance imaging (fMRI) experiment.

Schematic description of the procedure of the fMRI experiment is illustrated in Figure 3. There were five runs for the whole fMRI design, with three musical runs alternating with two linguistic (speech–melody) runs. The duration of each run was approximately 450 s. In the musical runs, participants were instructed to listen to famous symphonies and concertos or atonal random sequences. Methods and results for these musical runs will be detailed in another article.

Auditory stimuli in the linguistic runs were delivered through scanner-compatible headphones at a volume sufficiently loud enough that participants could readily perceive the stimuli over the scanner noise. Eighty trials of the five conditions were presented in two linguistic runs in a pseudorandom order. Each trial began with a warning tone (2.8 kHz, 0.3 s). Then, the prime and target stimuli were sequentially presented. The participants were instructed to listen to them and to rate the similarity of their melodies by pressing a button (a right-most button for “very similar” and similarity-score of 4, a right button for “slightly similar” and similarity-score of 3, a left button for “slightly dissimilar” and similarity-score of 2, and a left-most button for “very dissimilar” and similarity-score of 1). This task was used to assess whether participants were attending to the task. In total, the pre-scan training session and the scanning session took approximately 85 min for each participant.

Twenty-four to twenty-seven months after the fMRI experiment, the participants were asked to fill out a short online questionnaire. Fourteen participants completed this questionnaire. In this online questionnaire, auditory stimuli of five trials of the ML-match condition and five trials of the NL condition were randomly selected and presented in a random order. The participants rated each of the utterance (target stimulus) using a sliding scale to indicate how speech-like or song-like it was. Then, they rated on a sliding scale how much they agreed or disagreed these two statements: ‘I paid more attention to the musical melodies of the utterances that were preceded by matched melodies, compared to those preceded by noise’; ‘I more tended to covertly imitate the utterances that were preceded by matched melodies, compared to those preceded by noise.’ The results of this questionnaire were expected to reveal how the melody prime affected the processing of the target utterance.

### 2.4. MRI Data Acquisition

For imaging data collection, participants were scanned using a 3T MR system (MAGNETOM Prisma, Siemens, Erlangen, Germany) and a 20-channel array head coil at the Imaging Center for Integrated Body, Mind, and Culture Research, National Taiwan University. In the functional scanning, about 2.5 mm slices of axial images were acquired using a gradient echo planar imaging (EPI) with the following parameters: time to repetition = 2500 ms, echo time = 30 ms, flip angle = 87°, in-plane field of view = 192 × 192 mm, and acquisition matrix = 78 × 78 × 45 to cover whole cerebral area. For spatial individual-to-template normalization in preprocessing, a Magnetization Prepared Rapid Gradient Echo T1-weighted imaging with spatial resolution of 0.9 mm isotropic was acquired for each participant.

### 2.5. Data Analyses

One-sample one-tailed *t*-tests were used to determine whether each participant’s ratings of prime-target similarity for the 20 trials in ML-match were significantly higher than the chance-level score of 2.5. Participants were excluded from analyses of fMRI data if their ratings for ML-match were not significantly higher than this chance-level score. For the final sample included in fMRI analyses, paired-sample *t*-tests were performed to assess differences in the similarity ratings between ML-match and ML-mismatch, as well as between MM-match and MM-mismatch. For the ratings of speech-like or song-like traits of the target stimuli in ML-match and NL, a paired-sample two-tailed *t*-test was used to assess the effect of the melody prime. For the ratings of participants’ agreement with the two statements about auditory attention and subvocal imitation, one-sample two-tailed *t*-tests were used to determine whether these ratings were significantly greater than the neutral midpoint of the scale (neither agreed nor disagreed).

Preprocessing and analyses of the fMRI data were performed using SPM12 (Wellcome Trust Centre for Neuroimaging, LDN, United Kingdom). The first four volumes of each run were discarded to allow for magnetic saturation effects. The remaining functional images were corrected for head movement artifacts and timing differences in slice acquisitions. Preprocessed functional images were coregistered to the individual’s anatomical image, normalized to the standard Montreal Neurological Institute (MNI) brain template, and resampled to a 2-mm isotropic voxel size. Normalized images were spatially smoothed using a Gaussian kernel of 6-mm full width at half maximum to accommodate any anatomical variability across participants.

We performed an event-related analysis to recover the response evoked by each target stimulus. Statistical inference was based on a random effect approach at two levels. The data of each participant were analyzed using the general linear model via fitting the time series data with the canonical hemodynamic response function (HRF) modeled at the event (target). Linear contrasts were computed to characterize responses of interest, averaging across fMRI runs. The group-level analysis consisted of two paired *t*-tests for (1) the contrast of ML-match minus NL, and (2) the contrast of ML-match minus MM-match. We then identified regions that were significantly active for both the ML-match minus NL contrast and the ML-match minus MM-match contrast. This was done because both ML-match and MM-match involved pitch processing and tonal working memory processing, as the melody of the prime needed to be stored and compared to that of the target. To reveal activation clusters related to the perceptual transformation from speech to song across a musical prime, we applied an inclusive mask of the ML-match minus MM-match contrast on the ML-match minus NL contrast. In the fMRI analyses, statistical significance was thresholded at FDR-corrected *p* < 0.05 with a minimum cluster size of 10 voxels.

## 3. Results

**Figure 4:**
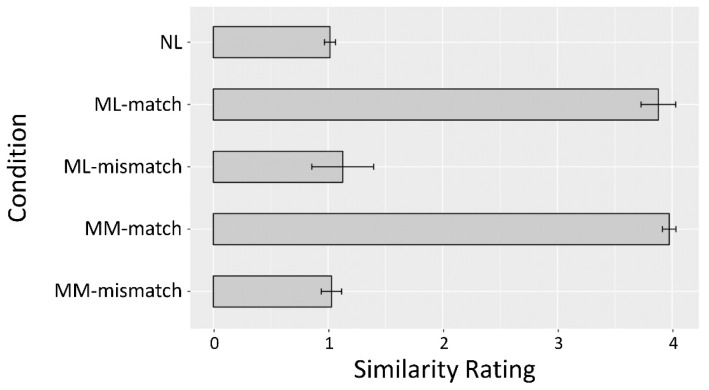
Participants’ ratings of prime-target similarity for five conditions. Error bars indicate standard deviation.

Analysis of the subjective ratings of prime-target similarity showed that one participant’s ratings for ML-match were not significantly higher than chance level (*p* = 0.44). This participant was excluded from the analyses of fMRI data because she was unable to discriminate between matched and mismatched trials at better than chance levels. The data of another participant were discarded because of incomplete behavioral data acquisition during the fMRI session. The final sample for fMRI analyses was therefore 18 participants, whose similarity ratings for ML-match were significantly higher than the chance-level score (*p* < 0.001). Figure 4 displays their rating data for five conditions. The similarity ratings for ML-match were significantly higher than ML-mismatch (*p* < 0.0001), and those for MM-match were significantly higher than MM-mismatch (*p* < 0.0001).

**Figure 5:**
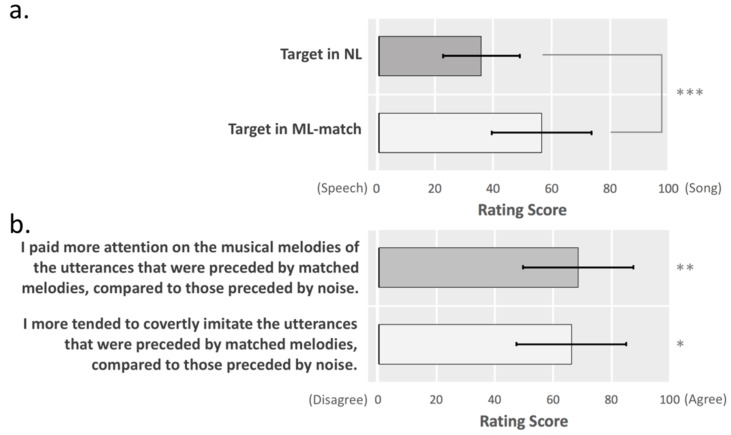
Results of the online questionnaire after the fMRI experiment. (**a**) Rating scores of speech-like or song-like traits of the target stimuli in NL and ML-match showed that the target stimuli in ML-match were perceived as more song-like than those in NL. (**b**) Rating scores of participants’ agreement with the two statements showed that the participants paid more attention to the musical melodies of the utterances that were preceded by matched melodies and more tended to covertly imitate these utterances, compared to the target utterances that were preceded by noise. Error bars indicate standard deviation. Note: * *p* < 0.01, ** *p* < 0.005, *** *p* < 0.001.

Analysis of the ratings of speech-like or song-like traits of the target stimuli in ML-match and NL showed that the target stimuli in ML-match were perceived as significantly more song-like than those in NL (*p* < 0.001). Analysis of the ratings of participants’ agreement with the two statements showed that the participants paid significantly more attention to the musical melodies of the utterances that were preceded by matched melodies, compared to those preceded by noise (*p* < 0.005). The participants significantly more tended to covertly imitate the utterances that were preceded by matched melodies, compared to those preceded by noise (*p* < 0.01), as shown in Figure 5.

**Figure 6:**
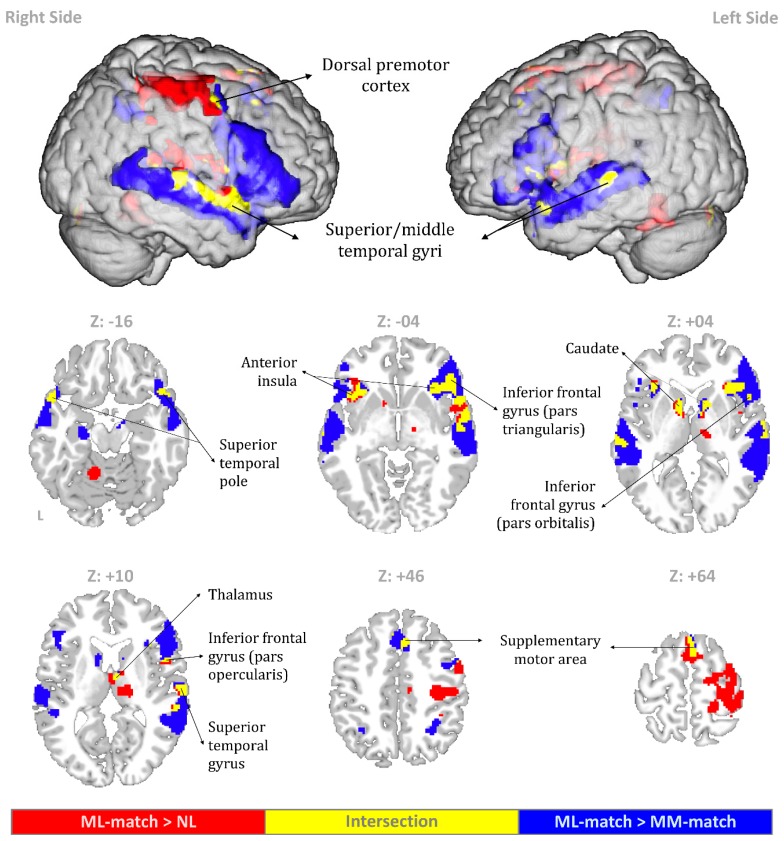
Group-level activation maps for ML-match minus NL (red), ML-match minus MM-match (blue), and their intersection (yellow).

Results of the whole-brain analyses of fMRI data were summarized by Table 1, Table 2, and Figure 6. Compared to the NL condition, the ML-match condition was associated with significantly increased activity in a number of regions, including the motor/premotor cortex, superior parietal areas, Rolandic operculum, temporal pole, anterior insula, IFG, superior/middle temporal gyrus (STG/MTG), SMA, caudate, thalamus, and cerebellum. Compared to the MM-match condition, the ML-match condition was associated with significantly increased activity in STG/MTG, temporal pole, IFG, anterior insula, superior parietal areas, hippocampus, SMA, putamen, caudate, and cerebellum. The intersection of ML-match minus NL and ML-match minus MM-match yielded activity in IFG, STG/MTG, dorsal premotor cortex, temporal pole, anterior insula, SMA, caudate, and thalamus.

## 4. Discussion

Spoken utterances in tonal languages are intrinsically rich in melodic content, and therefore the differentiation between speech and song in tonal languages is sometimes difficult to make. When a Mandarin speech-like utterance is preceded by a musical melody that mimics the speech melody, the listener may perceive this utterance as if it is being sung. In the present study, we used fMRI to explore this melody priming effect on pitch processing of Mandarin speech. Pitch contours of spoken utterances were modified so that the utterances can be perceived as either speech or song. Participants were asked to rate the melodic similarity between the prime and target. Analyses of fMRI data revealed increased activity in a number of regions for the intersection of speech preceded by matched music minus speech preceded by noise (ML-match > NL) and speech preceded by matched music minus music preceded by identical music (ML-match > MM-match), including the bilateral IFG, anterior insula, SMA, and STG/MTG. This finding echoes previous hypotheses and results of the speech-to-song illusion that exposure to repetition of a speech fragment is associated with greater activity in the neural substrates of pitch processing and re-evaluation of melodic features of this speech fragment [6,7,9].

The task of judging the melodic similarity between the prime and target in ML-match demanded the processing of melodic features of the target. Based on prior research on the neural correlates of prosody processing, we speculate that the right IFGtri, which showed activity for the intersection of ML-match minus NL and ML-match minus MM-match, may support the melodic processing of the speech-like target in ML-match. It has been reported that listening to “prosodic” speech (speech with no linguistic meaning, but retaining the slow prosodic modulations of speech) was associated with enhanced activity in the right IFGtri (extending into the pars opercularis of IFG) compared to normal speech [10]. The right IFGtri also responded to pitch patterns in song [16]. Moreover, a study of sarcasm comprehension in the auditory modality demonstrated that negative prosody incongruent with positive semantic content activated the right anterior insula extending into the IFGtri [12]. During perception of neutral, sad, and happy prosody, individuals with autism spectrum disorder displayed reduced activity in the right IFGtri compared to normal controls [10]. Taken together, we suggest that the right IFGtri may allocate attentional resources for the melodic processing of the target stimulus in ML-match. This view is supported by participants stating that they paid more attention to the melodic features of the target stimuli in ML-match, compared to those in NL.

One may speculate that the right IFGtri activity for ML-match minus MM-match reflects its role in working memory. However, both ML-match and MM-match involved a comparison of two melodies, a task demanding tonal working memory. One interpretation of increased activity in the right IFGtri for ML-match is that the task of melody comparison in ML-match preferentially relied on action-related sensorimotor coding of tonal information, whereas this coding played a lesser role in MM-match. There has been evidence indicating that the right IFGtri is engaged in the multi-modal processing of tonal or verbal information. For example, McCormick and colleagues investigated the neural basis of the crossmodal correspondence between auditory pitch and visuospatial elevation, finding a modulatory effect of pitch-elevation congruency on activity in the IFGtri and anterior insula [17]. Golfinopoulos and colleagues demonstrated that the right IFGtri exhibited increased activity when speech production was perturbed by unpredictably blocking subjects’ jaw movements [18]. Moreover, a study of sensory feedback to vocal motor control also reported that trained singers showed increased activation in the right IFGtri, anterior insula, and SMA in response to noise-masking [19]. This finding is especially relevant to our study, as we found co-activation of the right IFGtri, anterior insula, and SMA for the intersection of ML-match minus NL and ML-match minus MM-match. We suggest that the participants may use subvocal rehearsal to facilitate the task of melody comparison in ML-match. Indeed, our participants reported that they more tended to covertly imitate the target stimuli in ML-match compared to those in NL. During covert vocal imitation of the target stimulus, the anterior insula may be responsible for the laryngeal somatosensory functions and voice pitch control [20,21,22], the SMA may support motor planning and monitoring/evaluation of this plan [23,24,25,26,27], and the right IFGtri may allocate cognitive resources to integrate the auditory coding and action-related sensorimotor coding of the melodic pattern of the target.

Besides speech perception, the finding of the involvement of the bilateral STG/MTG in the melody priming effect on the pitch processing of Mandarin speech also provides an enriched perspective on song perception. Results of the online questionnaire showed that the target stimuli in ML-match were perceived as more song-like than those in NL. We found that the left mid-posterior STG/MTG was activated for the intersection of ML-match minus NL and ML-match minus MM-match. This cluster was effectively identical to that described by two previous neuroimaging studies on song perception. Sammler and colleagues reported mid-posterior STS activation for the interaction effect of lyrics and tunes during passive listening to unfamiliar songs, suggesting its role in the integrative processing of lyrics and tunes at prelexical, phonemic levels [14]. Alonso and colleagues reported that binding lyrics and tunes for the encoding of new songs was associated with the involvement of the bilateral mid-posterior MTG [15]. We suggest that the right STG/MTG may integrate the musical (melodic) and phonological information of the targets in ML-match. This view parallels an earlier report finding that STG/MTG was activated for the intersection of listening to sung words minus listening to “vocalize” (i.e., singing without words) and listening to sung words minus listening to speech [28].

A study of the speech-to-song illusion [9] showed a positive correlation between the subjective vividness of this illusion and activity in the pars orbitalis of the bilateral IFG, which also exhibited activation for the intersection of ML-match minus NL and ML-match minus MM-match in the present study. These regions have been implicated in a broad range of cognitive processes, such as response inhibition [29,30], response selection [31], working memory [32,33], semantic processing [34,35], and prosody processing [36]. The pars orbitalis of the bilateral IFG appeared to contribute to certain high-level cognitive processes necessary for the melody-similarity-judgment task. Its exact role remains to be specified by future research.

A few limitations of the present study should be noted. First, in the speech-to-song illusion [6] a spoken phrase was repeated without modification, whereas we modified the pitch contours of spoken Mandarin utterances so that they differed from normal speech. Caution should be exercised when comparison is made between the results of this study and those of the speech-to-song illusion. Future research could explore how the manipulations of pitch flattening and the clarity of tonality of spoken utterances impact the melody priming effect of on the pitch processing of Mandarin speech. It is also interesting to examine whether native speakers, non-native speakers (second language speakers), and non-speakers differ in the pitch processing of “songified” speech. A previous study compared speech-to-song illusions in tonal and non-tonal language speakers, finding that both non-tonal native language and inability to understand the speech stream as a verbal message predicted the speech-to-song illusion [37]. Second, the final sample included in fMRI analyses mainly consisted of amateur musicians. We cannot ascertain whether this melody priming effect can also be observed in non-musicians. Specific musical or cognitive abilities may correlate with the tendency of perceptual transformation from Mandarin speech to song. However, this idea remains to be tested in future studies.

## 5. Conclusions

The present study has examined the neural underpinnings of the perceptual transformation from modified Mandarin speech to song across a musical prime that mimics the melody of speech. Based on our fMRI data and previous literature, we suggest that the right IFGtri may play a role in allocation of attentional resources to the multi-modal processing of the melodic pattern of this stimulus. Moreover, the STG/MTG may integrate its phonological and musical (melodic) information. While these findings corroborate and extend previous studies on the speech-to-song illusion, we believe that further exploration of the melodic characteristics of tonal and non-tonal languages would significantly advance our understanding of the relationship between speech and song.

## Figures and Tables

**Table 1:** Activation clusters for the contrasts of ML-match minus NL and ML-match minus MM-match. (MNI: Montreal Neurological Institute; ML-match: melody-language-match; ML-mismatch: melody-language-mismatch; MM-match: melody-melody-match; MM-mismatch: melody-melody-mismatch; NL: noise-language).

Volume Information	MNI Coordinate			t-Value	Cluster (voxel)
	X	Y	Z		
**ML-match minus NL**					
Precentral gyrus	38	−24	52	11.81	1979
Postcentral gyrus	44	−32	62	8.53	
Dorsal premotor cortex	38	−2	64	7.26	
Inferior parietal lobule	36	−42	52	5.86	
Dorsal premotor cortex	54	−2	50	5.14	
Thalamus	14	−18	10	8.08	132
Superior temporal pole	62	6	−6	7.24	1310
Anterior insula	40	24	0	7.01	
Superior temporal gyrus	68	−18	6	6.65	
Inferior frontal gyrus (pars orbitalis)	46	20	−12	6.19	
Rolandic Operculum	46	−20	20	6.14	
Inferior frontal gyrus (pars triangularis)	48	32	−2	6.04	
Caudate	−10	8	2	6.87	260
Thalamus	4	−8	10	4.98	
Anterior insula	−34	20	0	6.31	365
Inferior frontal gyrus (pars orbitalis)	−42	18	−8	5.87	
Inferior frontal gyrus (pars triangularis)	−32	30	6	5.62	
Supplementary motor area	4	10	64	6.12	158
Cerebellar lobule IV–V	−12	−52	−20	5.76	375
Cerebellar lobule VI	−22	−52	−22	5.3	
Middle temporal gyrus	−68	−22	2	5.43	77
Superior temporal gyrus	56	−36	14	5.34	80
Cerebellar lobule Crus II	−4	−84	−30	4.79	39
Supplementary motor area	4	−16	56	4.68	91
Supplementary motor area	6	22	46	4.62	116
Rolandic operculum	48	6	10	4.5	43
**ML-match minus MM-match**					
Superior temporal gyrus	62	−4	−10	11.83	6545
Middle temporal gyrus	64	−24	−4	9.86	
Inferior frontal gyrus (pars triangularis)	48	30	−2	8.31	
Inferior frontal gyrus (pars orbitalis)	40	26	−8	8.20	
Anterior insula	46	20	−12	8.05	
Superior/middle temporal pole	50	14	−24	7.69	
Middle temporal gyrus	−58	−6	−10	11.58	3274
Superior temporal gyrus	−54	−12	−4	11.19	
Anterior insula	−32	20	0	6.73	
Inferior frontal gyrus (pars orbitalis)	−48	36	−10	6.23	
Inferior frontal gyrus (pars triangularis)	−46	30	10	5.49	
Hippocampus	−20	−18	−16	5.98	68
Supplementary motor area	4	22	48	5.52	359
Supragenual anterior cingulate cortex	−4	30	42	3.99	
Putamen	16	8	2	5.26	123
Thalamus	6	−6	10	3.95	
Caudate	−12	10	12	4.98	95
Inferior parietal lobule	34	−48	44	4.87	194
Angular gyrus	32	−58	48	3.79	
Parahippocampal gyrus	14	−2	−20	4.68	49
Cerebellar lobule Crus II	0	−84	−22	4.00	35
Superior parietal lobule	−32	−60	54	3.92	41
Inferior parietal lobule	−30	−58	42	3.59	

**Table 2:** Activation clusters for the intersection of ML-match minus NL and ML-match minus MM-match.

Volume Information	MNI Coordinate			t-Value	Cluster (voxel)
	X	Y	Z		
Middle temporal gyrus	−62	−22	4	10.98	77
Superior temporal gyrus	−58	−26	2	7.95	
Superior/middle temporal gyrus	66	−8	−2	10.44	1017
Inferior frontal gyrus (pars orbitalis)	40	26	−8	8.20	
Anterior insula	34	24	−4	7.88	
Inferior frontal gyrus (pars triangularis extending into pars opercularis)	52	16	6	7.45	
Superior temporal pole	60	10	−10	6.58	
Supplementary motor area Supragenual anterior cingulate cortex	4	22	48	5.52	61
	4	8	62	3.23	38
	−4	30	42	3.99	13
Superior temporal pole, superior temporal gyrus	−52	14	−20	5.31	238
Anterior insula	−32	20	0	4.64	
Inferior frontal gyrus (pars orbitalis)	−44	20	−6	3.34	
Caudate	16	8	2	5.26	63
Thalamus	4	−8	10	3.95	
Caudate	−8	0	4	4.98	48
Superior temporal gyrus	56	−36	10	4.63	28
Inferior frontal gyrus (pars opercularis)	50	8	8	4.46	18
Dorsal premotor cortex	48	2	52	4.39	29
Cerebellar lobule Crus II	−6	−84	−32	4.00	23

## Supplementary Materials

Audio examples of the stimuli for NL and ML-match are available online at https://www.mdpi.com/2076-3425/9/10/286/s1.
